# The Role of Talent Management Comparing Medium-Sized and Large Companies – Major Challenges in Attracting and Retaining Talented Employees

**DOI:** 10.3389/fpsyg.2018.01750

**Published:** 2018-09-19

**Authors:** Eva Boštjančič, Zala Slana

**Affiliations:** Department of Psychology, Faculty of Arts, University of Ljubljana, Ljubljana, Slovenia

**Keywords:** talent management, attracting talent, retaining talent, practices, medium-sized companies, large companies

## Abstract

In order for companies to realize their organizational visions, they need staff who are high-potential and looking toward the future. Due to the demographic, social and economic situation in Europe, the labor market is already reflecting a lack of high-quality human resources (HR), which inspires research into and planned management of high-potential, i.e., talented, employees. Companies are aware that only those organizations that recognize this area as crucial and invest resources into it will be successful in the “war for talent.” The purpose of the study was to research the field of talent management from the perspective of the definition of what the talent management process means for companies, how to attract and recognize talented employees, what development activities to provide them with and how to measure their performance and progress. We employed an exploratory approach, using the method of semi-structured interviews to gather information from 21 HR professionals who work at medium-sized and large Slovenian companies. We found that these organizations use various approaches and activities to attract and develop talented employees. At two thirds of the companies, performance is measured using annual evaluation interviews, by measuring the meeting of targets and evaluations by superiors. The biggest challenges in the field are attracting talented employees and positioning the organization as a desirable employer. The study is useful primarily as an overview of the field and of best practices, which companies can use to argument their existing processes.

## Introduction

Human resources (HR) management professionals say that talent management, defined as the process through which organizations meet their needs for talent in strategic jobs (Cappelli and Keller, 2014), is one of the biggest challenges that organizations will face in the 21st century (cf. Ashton and Morton, 2005). Analyses indicate that talented employees make up from 3 to 5% of all employees within an organization (cf. Berger and Berger, 2004; Nikravan, 2011). In view of its current popularity and the relatively large amount of past research one might expect the field to be well defined and supported by a wide range of research and praxis. However, numerous writers and researchers believe that the field currently lacks a clear definition, framework and general objectives (Lewis and Heckman, 2006; Cappelli and Keller, 2014), a stable theoretical basis (Thunnissen, 2016) and empirical research (Collings and Mellahi, 2009; Cappelli and Keller, 2014). Gallardo-Gallardo and Thunnissen (2016) found that the largest amount of empirical research on talent management is conducted in the United States, Great Britain, Australia, Netherlands and Ireland. This has led to a noticeable Anglo-Saxon influence and a focus on researching the process of talent management in the private sector and in multinationals (Thunnissen et al., 2013). As a result, the concepts and practices based on this literature can not be fully transferable to other types of organizations, such as medium-sized and large (non-multinational) companies, or to other cultures.

In addition to growing interest in research in the field of talent management, the labor market has also changed considerably in the last 10 years (Mucci et al., 2016) due to different social and economic changes (Giorgi et al., 2015). The global economic crisis that engulfed Slovenia in 2008 and only began to recede in 2015 (Kohont and Stanojevič, 2017) had a major impact on the Slovenian economy and consequently on the labor market and HR management – through layoffs, by a reduction in hiring or increased use of flexible types of labor (Todorovič Jemec and Vodopivec, 2016). Along with Cappelli and Keller’s literature review (2014) we will try to analyze basic challenges associated with selecting and managing talents in modern labor market. Therefore for purposes of the present paper we were interested in how medium-sized and large enterprises in Slovenia approach the talent management process. The first aim of this research is an overview of how Slovenian companies describe talent management, and the second aim is drawing parallels and differences in the process between medium and large sized companies. The answers to the given questions and an initial review of the literature will represent the starting point for a better understanding of the talent management process in the Slovenian and the broader European context.

### Lack of Clear Definition – What Is Talent Management and What Does Talent Even Mean

The majority of definitions define talent management as a comprehensive, well-planned and systematic process that includes attracting, identifying, selecting, developing, and assessing talented employees, in order to increase efficiency of operations (cf. Automatic Data Processing Inc [ADP], 2011). In comparison to HR management, practitioners say that talent management covers more narrow scope, that it emphasizes segmentation rather than egalitarianism, that it focuses on people rather on function and stresses the capture and retention of talents (Iles et al., 2010).

Some definitions mention an inclusive approach – the development of all employees so that they achieve their highest potentials (cf. Ashton and Morton, 2005), where some experts (Ariss et al., 2014; Cappelli and Keller, 2014) describe talent management as the development and posting of employees or jobs who are critical to the success of the company – the exclusive approach or the strengths-based approach. Inclusive approaches have been developed more recently, as well as workplace regulations requiring equal treatment of employees.

The definitions also vary with respect to how many areas they include under the concept of talent management; they can be divided into three groups – the first sees talent management merely as human resource management, predicting demand and personnel planning (cf. Cappelli, 2008), which on a practical level is merely a different formulation of the activities usually performed by HR. The second group of definitions refers to succession planning and creating a flow among jobs within the organization (cf. Warg, 2008), while the third is entirely focused on attracting, recognizing and retaining talented employees, the identification of key strategic positions and the creation of a pool of talented employees to fill them. There is still opened question about the borders between HR management and talent management.The definition of the concept of talent is also unclear – is it just a new name for an already known construct or group of constructs (e.g., HR; Cappelli and Keller, 2014), or is it something else? A common understanding is that talent refers to those employees who are identified as having the potential to reach high levels of achievement (Tansley, 2011). The word talented could be compared with its synonyms – e.g., gifted (cf. Chamorro-Premuzic and Furnham, 2010), highly capable, genius, extraordinary, exceptional, above average, etc., All of these descriptions of talent can be divided into four subcategories – aptitude, creative thinking, social intelligence and task orientation. Research on the definition of talent has also focused on the specific skills needed to guide and manage talented employees, as they are oriented toward recognizing potential in employees so that they will be able to take key strategic (often leadership) positions within the company. Claussen et al. (2014) identified the characteristics required for effective management into four areas: experience, expertise, social capital, and social network. Today an increasing number of organizations define talent based on the competence model that the company uses to define the important skills for an individual position (Reilly, 2008). There are also more organizations that are putting organizational values at the forefront, and in recognizing talented employees focus on the level of fit between the employee and the organization. This raises the question of whether employees who are recognized as talented in one organization can succeed at another, despite the fact that the skills or values might be different.

### Attracting and Retaining Talent

One of the most important concepts relating to attracting and retaining talented employees is the corporate brand, as its qualities and popularity increase the perception of its value (Wallace et al., 2012). Job selection contains a high level of risk, which motivates potential job candidates to invest their time and energy into searching for information about their potential employer. Signal theory (Vickrey, 1961) says that in order to avoid making bad decisions, people rely on signals which make it easier for us to form opinions about quality, so organizations have to use their corporate brand to communicate appropriate signals and thus increase the possibility that candidates will attribute a competitive advantage to it.

Many companies when planning talent management think first of all about attracting talented individuals from the labor market, but pay less attention to using scientifically proven methods and approaches to recognizing talent (Pulakos, 2005). A study conducted in Germany (Benit et al., 2014) indicated that when selecting employees companies seldomly employ quality-oriented methods such as annual evaluation discussions, psychometric testing or evaluation centers. Some organizations use a 9-box grid to identify “high-potentials,” who are described as competent, engaged and striving to become leaders, and “high-performers,” who have already demonstrated such skills, viewpoints and behaviors.

Human Resources personnel frequently question the rationality of whether or not to notify employees about being classified as talented. Ready et al. (2010) find that 85% of organizations communicate this transparently. A study conducted by Church et al. (2015) supplements this data, finding that only 34% formally provide information about the status of their employees regarding classification as talented, but that individuals who are informed about their talented status are more loyal to the organization – just 14% seek opportunities elsewhere.

### Retaining Talented Employees Is Closely Linked to Talent Development

During economic booms companies face increased fluctuation, which brings to the fore the question of how to create conditions so that the best employees are retained. One of the possible answers is to provide education and training and skills development opportunities for talented employees. The challenge is to connect all of the important parts in a systematic manner that allows the development of the individual’s competences and at the same time indicates their performance. First the individual has to be analyzed – their experience, knowledge, skills and qualities – and then a work plan can be developed based on their strengths and weaknesses and the desired competences for a specific position or generally within the organization. It is important to stick to the plan, create an optimal environment for learning, create feedback loops from both sides, etc., (Berger and Berger, 2004). Organizations use numerous methods to develop talent, e.g.: coaching, job rotation, problem-solving meetings, assuming leadership in emergencies or replacing absent staff, participation in project groups or working on special projects, corporate universities, workshops and training courses, guided reading and guided discussions, extracurricular activities, mentorship, e-learning, and job shadowing.

Another activity carried out within talent management is *succession planning* – developing a pool of individuals whose advancement is planned (Rothwell, 2011). Succession planning is a process in which needs are identified at various levels, key positions are identified and talent pools are created for each level. For each individual, in addition to their individual plan it is necessary to think about how much time it will take to develop their skills to a certain level – i.e., how much time they will need to progress and actually assume a higher position. A study conducted in 2006 (Fegley, 2006) indicated that just 6% of organizations are “extremely prepared” to fill their leadership positions, 53% described their situation as “prepared,” 37% as “unprepared” and 4% as “extremely unprepared.”

### Performance Appraisal – How to Measure the Effectiveness of Talented Employees

A review of the literature does not indicate any particular differences in the measuring of effectiveness between employees and high-potentials, i.e., talented employees. In practice, organizations usually measure the effectiveness of high-potentials through performance appraisal interviews – superiors assess various aspects of the employees’ performance and offer them feedback on their performance evaluations (DeNisi and Murphy, 2017). When measuring work effectiveness, the majority of organizations focus on the reaching of performance goals, which they assess using key performance indicators (KPIs). This approach is associated with management by objectives, in which superiors and subordinates agree on work duties and responsibilities for a certain period, define the specific targets, measure those targets and set out a time frame (Gladisa and Susanty, 2018). Trends are moving toward identification of competencies as part as the performance management. Development appraisal could be incorporated in the performance appraisal, but literature review shows that many firms are not appraising competencies in the performance appraisal process (e.g., Abraham et al., 2001).

### Comparing TM Practices in Medium-Sized and Large Companies

In Slovenia, organizations with 50 to 249 employees are considered medium-sized, while those with over 250 employees are considered large (Uradni List Republike Slovenije, 2006). In addition to the number of employees, these companies also differ with regard to other institutional, economic and structural characteristics which are reflected in the talent management process.

The majority of studies have focused on large multinational enterprises, and the concepts and practices from that environment are frequently uncritically transferred to medium-sized enterprises. Medium-sized enterprises more seldomly focus on the recognition of key positions, as these change as the organization grows, while the identification of key employees who are effective and flexible and who can take on various roles as the organization grows plays an important role (Krishnan and Scullion, 2017). Growth causes increased unpredictability, and the organization has to maintain a certain level of flexibility to counter this, which makes it more difficult to implement planned and systematic staffing processes. Medium-sized enterprises are also characterized by a higher level of informality, which is usually associated with a more personal management style – thus talent management practices are often carried out on a more unplanned, unsystematic, intuitive level. Other companies practice talent management and carry out activities that are generally found within that process, but do not label them as talent management (Valverde et al., 2013).

The size, duties and functions of the HR department also depend on the size of the company. The HR departments in medium-sized enterprises are usually small, with individual employees covering several areas, and they sometimes have a more operative rather than a strategic function. The average number of employees in medium-sized enterprises is 100 (EU data), which is also the size at which companies start thinking about introducing specialized HR positions (Valverde et al., 2013).

Studies (e.g., Ready et al., 2010; Krishnan and Scullion, 2017) indicate that the exclusive approach predominates at large companies (recognition of handfuls of high-potential/key employees and targeted management of them), while medium-sized enterprises usually use an inclusive approach, in which talent management activities are targeted toward all employees. A German study (Festing et al., 2013) found that 54% of small and medium-sized enterprises include all employees in the talent management process, 29% focus on technical experts as the most important group, 10% direct activities toward experienced managers and the board of directors, and 8% to high-potential employees.

In view of the above findings, we decided to conduct exploratory research with semi-structured interviews in order to obtain an insight into the practice of talent management in medium-sized and large enterprises (with respect to the number of employees). We wished to analyze how organizations in Slovenia define the talent management process, how the process itself proceeds (attraction, recognition and development of talented employees), how effectiveness is measured, and the future development guidelines in this area. We foresaw that we would obtain the largest amount of information on this from the HR professionals who represented our participants. Through a review of the literature and practices we want to contribute to the understanding of the talent management process in Slovenia and in the broader European context.

## Materials and Methods

### Participants and Procedure

The study included 21 employees who work in the field of talent management in medium-sized and large enterprises in Slovenia. They included 16 heads of HR departments, two personnel development specialists, a regional HR generalist, an HR specialist and a psychologist. The sample included 13 large companies with over 250 employees, and eight medium-sized companies with 50 to 250 employees (in 2016 there were 339 large companies and 2027 medium-sized companies registered in Slovenia). The largest number of the companies in our sample work in the processing industries (*N* = 11), followed by the financial and insurance industry (*N* = 4), the information and communications industry (*N* = 3), motor vehicle sales, maintenance and repair (*N* = 2) and electrical, gas and steam power generation (*N* = 1).

We conducted semi-structured interviews lasting from 22 to 67 min. We made audio recordings of all of the interviews so that they could be transcribed and analyzed at a later time. The participants first signed informed consent forms for participation in the study, in which they were notified of the responsible persons (contact data), the purpose of the research, a description of the duties and requirements of the participants, the duration of their participation, any compensation, dangers and benefits, the voluntary nature of participation and protection of privacy. The interviews were conducted by a psychologist who guaranteed the anonymity of the participants. Sampling was conducted using the snowball method, as the researchers posted the invitation to participate on social and corporate networks, via their own social networks and electronic mailing lists. Only one restriction was included in the invitation, as we wished to restrict the sample only to employees of medium-sized and large Slovenian enterprises. We contacted a total of 50 organizations, of which nine refused the invitation, stating that they did not wish to participate because of time constraints (*N* = 6), they do not engage in personnel development or talent management processes (*N* = 2) or they do not see any advantage of participating in the study for their own organization (*N* = 1), while 20 organizations did not respond to the original invitation. We also decided to stop sampling after 21 participants, as we reached the theoretical saturation point (informational redundancy), meaning that the responses from later interviews became more predictable and no longer created added value (Fusch and Ness, 2015).

### Methods and Analyses

In view of the lack of research into talent management in Slovenia, particularly in the case of medium-sized and large enterprises, we selected an exploratory approach. We used the qualitative method for collecting data in the form of individual semi-structured interviews, in which the emphasis was placed on an in-depth understanding, explanation and description of the talent management process, and not on determining frequencies, quantities or qualities. This approach is inductive and interpretive, since the goal is to research the process and the formation of concepts.

We drew up eight initial questions within five areas, on the definition of the talent management process, the definition of the concept of talent, attracting talent, recognizing talent, transparency of communication, the development of talented employees, measuring the effectiveness of talented employees and the challenges in this area in the future.

The audio recordings were transcribed into literal transcriptions of the interviews. We used two basic qualitative techniques for the further analysis of the data from the transcriptions: coding and categorization. In coding we assigned a keyword to a certain part of the text for later identification of that part of the text. Later we used simultaneous coding, if two or more codes carried single information. Only for question on how they define talent management, we used structural coding, because we already predicted different groups of definitions (e.g., exclusive/inclusive) based on literature review. The next step was categorization, in which we placed the codes into a hierarchical structure, i.e., categories. Finally we conducted a comparative analysis in which company size was selected as the basis of comparison and the processes at medium-sized and large enterprises were compared. We also employed interpretation, where we attempted to place the phenomena into a broader context (Saldaña, 2016).

## Results

### Definition of Talented Employees and the Talent Management Process

Definition of talent, gathered from answers of our participants (**Table 1**), consists of acting in line with organization’s values, positive personality features, above average job performance and results, positive work related behaviors and intellectual capacity. More detailed revision of the answers is stated below.

**Table 1:** Definition of talent by categories of participants’ answers.

Categories	Subcategories	Pct.
Acting in line with organization’s values		43
Personality features	Willingness to learn	33
	Rapid acquisition of new knowledge	33
	Motivation	33
	Desire for development	33
	Healthy ambition	29
	Self-initiative	29
	Proactiveness	29
	Curiosity	29
	Willingness to share knowledge	14
	Team orientation	14
	Cooperation	14
Above average job performance and results		29
Positive work-related behavior	Makes proposals	19
	Makes suggestions for improvements	19
	Is capable to implement improvements	19
	Puts additional effort in order to do the work (i.e., go extra mile)	19
Intellectual capacity		14

In comparison, participants from one large and two medium-sized companies believe that personal qualities such as knowledge are more important, while participants from two large companies emphasized experience and expertise.

To the question of how companies define the talent management process and how they define the concept of talent, 13 participants (62%) defined talent management as an exclusive approach. Head of HR, large company: *“We have three groups of employees: key staff, high-potentials and young high-potentials.”* However, the remaining eight participants (38%) defined the process as inclusive: *“…we manage all of our employees in the same way – all of them have talents that they can develop.”* The head of HR at a medium-sized company used a new expression. *“Instead of* talent management *we call the field* giving perspective.”

With regard to the three streams of thought relating to talent management (Lewis and Heckman, 2006), the majority of the definitions we collected (*N* = 10) are associated with the stream which is entirely focused on the management of talented employees. Thus the head of HR at a large company defines talent management as *“… a series of activities that starts with attracting and continues with identification and development of talented employees. Nowadays retaining staff is becoming increasingly important…”* Participants from seven organizations emphasize succession planning, which can be summarized with the definition *“Talented employees are our key staff and their potential replacements.”* The lowest number of companies (*N* = 4) equated talent management with human resource management, with the head of HR at a medium-sized company stating that: *“Part of the duties of the HR department is to ensure the development of its employees.”* If we view the talent management process through the lens of employees at medium-sized and large companies we find that nine large companies understand the process as exclusive and four as inclusive, while this ratio is even among medium-sized companies (*N_excl_* = 4, *N_incl_* = 4).

During the interviews we also asked the participants additional initial subordinate questions about the number of recognized talented employees, the duration of planned talent management activities at the company and any obstacles to working in this field. The percentage of talented employees within the organizations is difficult to define, as some companies include key staff (management and professionals) in this group. In contrast, some companies cannot supply this data since they manage talent inclusively and do not separate employees into talent brackets. At companies that had data available, 7% of employees on average are recognized as talented (*Max* = 20%, *Min* = 1%). When comparing medium-sized and large companies, medium-sized companies have a higher average number of recognized talented individuals (*M* = 11%) than large companies (*M* = 4%).

The participants from six companies stated that they began the planned development of the field of talent management in 2017, despite the fact that the initial stages of the process go back several years. At that time they started focusing on the field more intensively and in a more planned manner, and also started communicating about it more. Five companies began introducing talent management and a succession system between 2010 and 2014, while one large company reported a longer period of involvement reaching back to a project in 2008. Some (*N* = 3) participants were unable to respond to the question, as they joined their companies later and did not know exactly when their company had initiated the talent management process.

Among limitations to the selection of talented employees, some participants noted that they seek talented employees via internal job listings with prior exclusion, in which the most frequently mentioned categories are age (e.g., up to 40 or 50), education level (e.g., at least high-school, others post-secondary or university education) or seniority within the company (e.g., at least 1 year or at least 2 years). Other companies have defined various groups of talented employees (e.g., talented managers, talented professionals and talented young employees), which includes the group of high-potentials with an age limit of up to 33 or 35. Some also see young employees who are hired immediately out of college as talented employees.

### Attracting, Recognizing, and Communicating About Talented Employees

The largest number of companies (48%, *N* = 10) attract talented employees through scholarships, for both college and high-school students, planned brand management and cooperation with universities (e.g., employees occasionally lecture at universities, professors recommend students). They attend job fairs (38%, *N* = 8), and allow students to gain experience during their school years through traineeships and apprenticeships (33%, *N* = 7), work with high schools (27%, *N* = 6), update job listings in order to increase the company’s attractiveness (24%, *N* = 5) and hold company open houses (19%, *N* = 4). Less common practices (14%, *N* = 3) include joining the BusinessClass program, mentorship for Bachelor’s and Master’s theses, the organization of business hackathon (event that brings people from different sectors together to tackle challenges) and working with career development centers. The participants also mentioned (10%, *N* = 2) occasional support for professional field trips for students, summer schools or extended educational programs for students. Six companies also mentioned special projects designed to attract specific groups of talented employees.

In order to recognize talented employees, the largest number of companies (38%, *N* = 8) use developmental interviews led by HR (at which the employee’s wishes and plans are discussed), and two companies encourage their employees to visit the HR department or a psychologist and recognize their talents collectively. The participants also mentioned taking into account the suggestions of managers (38%, *N* = 8) who identify talented employees in their departments and the information that they collect through the annual evaluation interviews (with respect to their job performance and performance over a longer period of time). Frequently, companies identify talented employees using the technique of calibration between management and HR (24%, *N* = 5), followed by calibration with the board of directors (19%, *N* = 4). A full 29% (6) of companies use psychological testing to identify talented employees, and 14% (3) also use employee evaluation centers. Nearly one fifth of companies (19%, *N* = 4) use workplace observation (observation during projects, teamwork, at meetings) and project evaluations (14%, *N* = 3). Some companies (14%, *N* = 3) organize the talent recognition process through applications which are open to all employees who meet the criteria, followed by a further selection process. Similarly, 14% (3) of companies recognize talented employees based on skills evaluation (using various versions of the 360-degree method). Other approaches, which are used by only a few companies, include: employee evaluations by managers (evaluation of skills or behavior in relation to corporate values), design and implementation of independent projects (more under Discussion), employee evaluation during the onboarding process, and an information system that collects data on employees and then makes talent appraisals (e.g., age, education, job performance, evaluations by superiors in various surveys, psychological tests, etc.).

After the identification of talented employees, the selected employees must be notified. Two thirds of participants (67%, *N* = 14) reported that they communicate transparently, 19% (*N* = 4) do not share such information with employees, 14% (*N* = 3) share only a part of such information (the employees are briefed on development plans and invited to participate in development activities, but their status is not communicated publicly or to other employees). If we compare large and medium-sized enterprises, 85% (*N* = 11) of large companies communicate the recognition of talented employees transparently, while only 38% (*N* = 3) of medium-sized companies do so.

### Development of Talented Employees

In the next section we were interested in the ways that companies **develop** talented employees. They selected the most commonly used methods and approaches from a list, and were required to give specific examples from their organizations.

In addition to the aforementioned methods and approaches (**Table 2**), talented employees are also afforded additional opportunities such as training abroad, mobility, inclusion in interdisciplinary teams, knowledge-sharing with other employees, inclusion in various external programs, connections to start-ups, meetings with members of the board of directors, and internal personal development programs.

**Table 2:** Methods for developing talented employees in the workplace.

Methods, approaches	Pct. (*N*)	Applications
Workshops and training courses	100 (21)	Leadership skills; Soft skills; Professional training
Working in project groups	91 (19)	Frequent inclusion of high-potentials in interesting projects; Invitations to apply on their own for inclusion in new projects
Problem-solving meetings	81 (17)	Use of the “design thinking” method; Implementation of quality teams; Meetings designed to evaluate solutions
Working on special projects	81 (17)	Employees themselves propose projects or solutions, and can implement them if appropriate; Final projects at corporate universities
Coaching	81 (17)	Mainly for management positions; Internal coaching for sales areas
Assuming leadership roles in emergencies and frequent replacement of managers	76 (16)	High-potentials replace managers during maternity leave; Assume work duties during managers’ vacation times
Job rotation	68 (14)	Encouraging internal mobility; Onboarding
Extracurricular activities	62 (13)	Co-funding of membership fees; Participation in expert committees; Charitable causes; Sports and arts and cultural societies
Mentorship	62 (13)	Professional/leadership mentoring; Education of mentor or mentee
E-learning	62 (13)	Access to global educational websites; Both technical content and soft skills
Succession planning	48 (10)	Planed inclusion of talented employees, or pool of successors and talented employees partially overlap
Job shadowing	24 (5)	Not just shadowing but also inclusion in work, mentoring
Corporate university	24 (5)	Working in modules; Cooperation with external educational institutions
Guided reading and guided discussions	14 (3)	Reading of professional literature and discussion at meetings

We were also interested in whether they have established succession plans and whether talented employees were included in them. Just under half of the companies (47.6%, *N* = 10) have a succession plan which includes talented employees. In the majority of cases the talent pools and the successor pools overlap, but not entirely. The other companies do not have succession plans, but say that it proceeds on a more intuitive level and non-systematically (38.1%, *N* = 8), or that they are planning in implementing one in the near future (14.3%, *N* = 3). A comparison of medium-sized and large companies indicates that a larger number of large companies have systematically determined succession (*N* = 7) than those that do not (*N* = 6), while only three medium-sized companies have planned succession, and five determine successors on an intuitive basis.

### Assessment of the Effectiveness of the Work of Talented Employees

The data indicate that two thirds of companies (66.7%, *N* = 14) measure employee effectiveness through annual evaluation interviews. The largest number of companies (42.9%, *N* = 9) use KPIs (quantitative or qualitative) to measure performance on a monthly, quarterly or annual basis. The same number of companies (42.9%, *N* = 9) measure job performance through the development of the individual’s skills, using the 360-degree method, evaluation centers or other tools. Targeted management is used at 38.1% of companies (*N* = 8), where the achievement of goals is checked periodically. One fourth of companies (23.8%, *N* = 5) entrust the assessments to managers who evaluate employee performance using various tools – questionnaires, scales or descriptive evaluations. Some companies check effectiveness via project evaluations (23.8%, *N* = 5), and at one of them this assessment is made by the heads of the various project groups, who each check certain targets. The following approaches are used less frequently: acting in accordance with values (superiors grade employees on a scale of behavioral descriptions of values), evaluation of effectiveness via proposals for promotion or actual promotions in a certain time period, informal opinions (co-workers, collaborators on projects, project leaders), self-evaluations, performance scores with respect to the performance of the company as a whole, performance scores with respect to the performance of the individual department as a group, number of proposals or ideas that the individual makes or has in a certain time period.

### Future of the Field of Talent Management

Human resources professionals see the greatest future challenges in the area of attracting personnel (*N* = 14), followed by retaining personnel (key staff, young high-potentials) (*N* = 6) and maintaining staff in organizations in which there is no possibility of advancement. They also listed challenges in the area of managing expectations (*N* = 2) and transparency in communicating the succession plan and talent pool system (*N* = 2). Individual participants see potential difficulties in the area of the objective recognition of potentials, the creation of a good model for developing potentials, measuring the effectiveness of the talent management process and personnel planning. They wonder how to balance the complexity of the talent management process and the time that has to be invested in it by managers and HR departments and for development activities for recognized employees.

## Discussion

In this study we have attempted to analyze the current situation in the area of talent management in medium-sized and large enterprises in Slovenia. We found a disparity among the various definitions of talent management in our sample, as more than half of the participants focused on the development of a specific group of people who are crucial to the success of the company (exclusive approach), while others emphasize the development of the strengths of all employees (inclusive approach). The results indicate a similar trend to the research of other authors (e.g., Ready et al., 2010; Cappelli and Keller, 2014; Krishnan and Scullion, 2017), since large companies more often employ the exclusive approach while medium-sized companies use both approaches equally. We estimate that the situation in Slovenia will change in the near future based on the obligatory implementation of regulations requiring equal treatment opportunities for all employees. With regard to the three different sets of concepts (Lewis and Heckman, 2006) the largest number of definitions focus on comprehensive management of talented employees (attracting, recognizing, and retaining), followed by those which prioritize succession planning, while some companies believe that talent management is merely a different formulation of the basic duties of the HR department – human resource management. Despite the belief of certain authors that talent is transferable between organizations (e.g., Chamorro-Premuzic and Furnham, 2010), the results indicate that in identifying talented employees, most companies prioritize corresponding to company values – talented employees are therefore those whose values correspond to the organizational culture. We can then describe the construct using a list of the qualities highlighted by HR professionals, at the top of which are: willingness to learn, rapid acquisition of new knowledge, motivation, a desire to develop, ambition, self-initiative, proactiveness, and curiosity. The majority of the participants agree that what is required is a combination of embodying the values, personal characteristics and above-average job performance over a certain time period.

The majority of the participating companies initiated a more planned and intensive approach to the talent management process after 2015, which also corresponds to the period of the abatement of the economic crisis (Kohont and Stanojevič, 2017). An HR specialist at a medium-sized company stated that the reason for this is that “… *in the time after the crisis talent management became a competitive advantage for companies that are fighting to attract talented employees*.” Furthermore, during this period some companies created more specialized development positions within their HR departments. The organizations have differing perspectives on age, educational and other limits when selecting talented employees. Large companies on average recognized 4% of their employees as talented, which is in line with the results of other studies (e.g., Berger and Berger, 2004; Nikravan, 2011). In our study the percentage of employees recognized as talented was higher at medium-sized companies (11%), which can be attributed to a broader definition of talented employees, which often include other groups, e.g., key employees.

The most common activities that companies reported in relation to **attracting talented employees** from the market were scholarships and cooperation with universities. Nearly half also employ planned development of the employer’s brand and thereby increase the possibility that candidates will see them as interesting and desirable – they adapt their selection process in order to make it more interesting, share stories of their employees’ successes, actively participate in professional events within their spheres and offer employees attractive packages (flexible work arrangements, additional benefits). The **recognition of talented current employees** is most often based on managers’ proposals and the expression of a desire to develop in interviews conducted by HR. Similar to the German study (Benit et al., 2014), a little less than one third of the participating companies use psychometric testing to identify talent, and even fewer use evaluation centers. Medium-sized companies report the use of a 9-box grid matrix, but the use is probably more appropriate at companies with fewer employees, as it could be extremely time-consuming at large companies. In general, medium-sized companies frequently mention that the recognition of talented employees is more informal, since they know their employees well, have a lot of contact with them, are able to observe them in various work situations and projects, and they are able to onboard in a less systematic, more informal manner. 82% of the companies communicate about the recognition of talented employees transparently, which correlates with the findings of Ready et al. (2010), who give a figure of 85%. Complete transparency includes giving feedback about the reasons for their (non-)inclusion in the talent pool, familiarization with the development plan and the presentation of talented employees at internal events. Companies that do not communicate this message to recognized employees state that the reason for this is that they do not wish to create expectations that they will not be able to meet in the future. At medium-sized companies the communication is usually less direct, and the underlying reasons for this are usually less systematic, more informal processes within which the detailed planning of career paths is more difficult. Regardless of the size of the company, it is above all important that this communication be carefully considered, since its effect can either be motivating or demotivating.

Development activities are another important factor in retaining key employees. Companies offer the majority of the abovementioned activities to all employees, while talented employees are involved in them more often and more intensively. More specific corporate practices also include training abroad, involvement in the work and activities of subsidiaries and the development of mobility through working at various organizational units. They also include talented employees in interdisciplinary teams so that they can obtain broader knowledge, and promote internal mobility so that people can develop their talents in the right positions. The companies state that retaining staff is important in order to broaden their positions and become more interdisciplinary. Around half of the companies also have a systematic succession plan, which is slightly lower than indicated in an American study (Fegley, 2006). Companies that do not plan succession as transparently argue that in a fast-changing business environment it does not make sense to predict the future and to promise positions. Succession planning at medium-sized companies is carried out on an intuitive level, which can be connected to the growth of the company and the changes to the organizational structure, whereby it is more important to recognize effective and flexible employees who could take on various roles.

In the majority of companies the measurement of the effectiveness of talented employees does not substantially differ from the measurement of the effectiveness of all employees. Annual performance appraisal interviews are used by two thirds of the companies in our sample. Companies usually use KPIs, skills evaluation using the 360-degree method and targeted management, while the majority of the assessments are based on the evaluations of superiors or self-evaluations. The head of the HR department at a large company noted that *“evaluation requires a combination – a matrix of job performance and a matrix of skills,”* while an HR specialist at a medium-sized company listed job performance (measurement based on achieving targets) and conduct in accordance with values as the most important factors.

The participants see the greatest challenges in attracting personnel, particularly from the point of view of positioning on the market as a desirable employer, attracting young candidates, staffing deficiencies and attracting people who are committed and passionate about their work and who work in accordance with company values. They feel it is important to present the corporate culture, work methods and organizational values as accurately as possible during the selection process, and for managers to internalize how important it is to engage with people. With regard to retaining personnel, a HR professional at a large company noted that the most important factor is *“getting the right people into the right positions at the right time.”*

The results of the study present another bridge between academic interest and practice (e.g., Cascio and Aguinis, 2008; Cappelli and Keller, 2014). A systematic review of the individual areas (attracting, recognizing, developing, and measuring effectiveness) can be applied comprehensively to the upgrading of existing processes within companies. The sharing of best practices can increase employee motivation and commitment, lower fluctuations and help companies design the process in a way that supports their vision and increases company performance. Similarly, cooperation between organizations in times of high fluctuation and brain-drains is also crucial for national development, so that Slovenia can position itself as a desirable country for people seeking employment.

One of the limitations of our study was the small sample size, which is difficult to extrapolate to all medium-sized and large companies. The sample also seems to be made up of companies that are perhaps not completely representative of the situation, as the study was proactively participated in by HR professionals at organizations with well-formed processes and which would like to share these practices with others. The results could also be affected by the participants’ subjective reporting, as we were not able to monitor the disparity between the projects the companies would like to implement and those that they actually have implemented, and the quality thereof. Additionally, Slovenia does have cultural and economic specificities, so a reader has to consider that when applying conclusions to other contexts.

In view of the fact that the participants in our study were Slovenian companies whose talent management activities are conducted mainly in a planned manner, it would also be valuable to analyze those companies that believe they do not engage in talent management and that decided not to participate in the study. In order to improve the research, the entire evaluation of the talent management process could be supported by a questionnaire that would be filled out by both talented employees, HR departments, senior management and the boards of directors. This would give us more reliable data on talent management in various areas and from various perspectives. Since our study focused on an analysis at the organizational level, in future research it would also be worthwhile to focus on the individual level, on the perspective of the talented employees, through a comparative analysis of the content planned by HR professionals, which is then implemented by talented employees and used for their own development.

## Conclusion

In this analysis we obtained an insight into the scope of the field of talent management at medium-sized and large companies in the period after the end of the recession. The results indicate that we are still unable to provide a uniform definition of the talent management process, as neither uniform definitions nor uniform practices exist. The concept of talent is defined as a combination of a fit with organizational values, personal qualities and job performance. The majority of companies have only been involved in the planned implementation of the talent management process for the last 3 years, which coincides with the end of the economic crisis. On average, 7% of employees are recognized as talented. Organizations attract talented employees through various activities and the planned development of the employer’s brand. Recognition is most often based on management proposals and through interviews, whereby medium-sized companies operate more informally in this respect. The majority of the companies are transparent in their communications with talented employees. They develop talented employees using various activities in which those employees are more intensively involved. Half of the companies have a systematic succession plan, whereby medium-sized companies carry out the planning at a more intuitive level due to the changing organizational structure occasioned by growth. Effectiveness is measured using annual discussions at two thirds of the companies, by measuring the achievement of targets and evaluations by superiors. HR professionals see the greatest challenges in attracting personnel and in positioning the company as a desirable employer.

The research involved cooperation of the Slovenian organizations, in which employers provide researchers access to detailed data in return for help generating a systematic review of the current areas and uncovered a number of unresolved issues. The findings can be used by HR professionals to upgrade their existing talent management processes, and offer opportunities for further research for professionals in the fields of psychology, economics, HR management and other areas.

## Ethics Statement

The administration adhered to the requirements of privacy and informed consent in the Slovene Law (Personal Data Protection Act 2004-01-3836 and subsequent amendments) and the ethical standards for research of the Declaration of Helsinki revised in Fortaleza (World Medical Association [WMA], 2013), followed and approved by the Department of Psychology of the University of Ljubljana (Slovenia). The administration adhered to the requirements of privacy in Slovenia law and informed consent was collected for each participant.

## Author Contributions

All authors listed have made a substantial, direct and intellectual contribution to the work, and approved it for publication.

## Conflict of Interest Statement

The authors declare that the research was conducted in the absence of any commercial or financial relationships that could be construed as a potential conflict of interest.
